# Two extremely rare cases of extrapleural hematoma

**DOI:** 10.1186/s40792-019-0760-0

**Published:** 2019-12-16

**Authors:** Soichi Oka, Kenji Ono, Kenta Kajiyama, Katsuma Yoshimatsu

**Affiliations:** Thoracic Surgery, Kokura Memorial Hospital, Asano, Kokurakita-ku, Kitakyushu-shi, Fukuoka, 802-8555 Japan

**Keywords:** Extrapleural hematoma, Hemothorax, Video-assisted thoracic surgery (VATS), Anticoagulation therapy

## Abstract

**Background:**

Extrapleural hematoma is uncommon. However, according to the size of hematoma and/or the progression of anemia, surgical treatment to control bleeding might be necessary because a huge hematoma can cause ventilator and circulatory disturbances to press heart and lung. We present two unusual cases of huge extrapleural hematoma in an anticoagulated patient with no apparent history of trauma or otherwise traumatic episodes.

**Case presentation:**

Case 1: A 78-year-old man presented to our emergency department with pain in his right shoulder and disturbance of consciousness. He had no apparent history of trauma. Computed tomography (CT) of the chest revealed the presence of a huge lens-like encapsulated lesion measuring 220 × 90 mm in the right thoracic cavity. These findings all supported a diagnosis of extrapleural hematoma with hemothorax. Case 2: A 73-year-old man was brought to our hospital by ambulance after bruising his back in his house. CT of the chest revealed the presence of a huge lens-like encapsulated lesion measuring 230 × 70 mm in the left thoracic cavity. Hemorrhagic effusion was obtained by thoracocentesis, and the lesion was suspected of being a hematoma. In both two cases, we performed video-assisted thoracic surgery (VATS), which was minimally invasive and effective. These two patients were cured and discharged smoothly after surgery.

**Conclusions:**

We reported two rare cases of extrapleural hematoma. This disease requires close attention when it manifests in patients undergoing anticoagulation therapy. Regarding treatment, VATS was particularly effective in these cases.

## Background

Extrapleural hematoma is uncommon. However, according to the size of hematoma and/or the progression of anemia, surgical treatment to control bleeding might be necessary because a huge hematoma can cause ventilator and circulatory disturbances to press heart and lung [1]. Poyraz et al. [2] recommended medical therapy or simple observation when the vital signs of an afflicted patient were stable and the hematoma was small; alternatively, surgical treatment was considered to be required if the hematoma was huge, causing circulatory and respiratory disturbances, or if the condition of the patient was unstable because of active bleeding. Therefore, the best approach for management of an extrapleural hematoma is controversial.

We herein report two cases of extrapleural hematoma and review the relevant literature.

## Case presentation

### Case 1

A 78-year-old man presented to our emergency department with pain in his right shoulder and disturbance of consciousness. He had no apparent history of trauma. His blood pressure was low (systolic blood pressure: 70 mmHg), oxygen saturation was 90%, and hemoglobin was 8.3 g/dl. His medical history included a giant vertebral artery aneurysm and coiling therapy performed 8 days ago, after which had been taking antiplatelet agents (aspirin and clopidogrel sulfate). Therefore, we considered that he was in hemorrhagic shock.

**Fig. 1:**
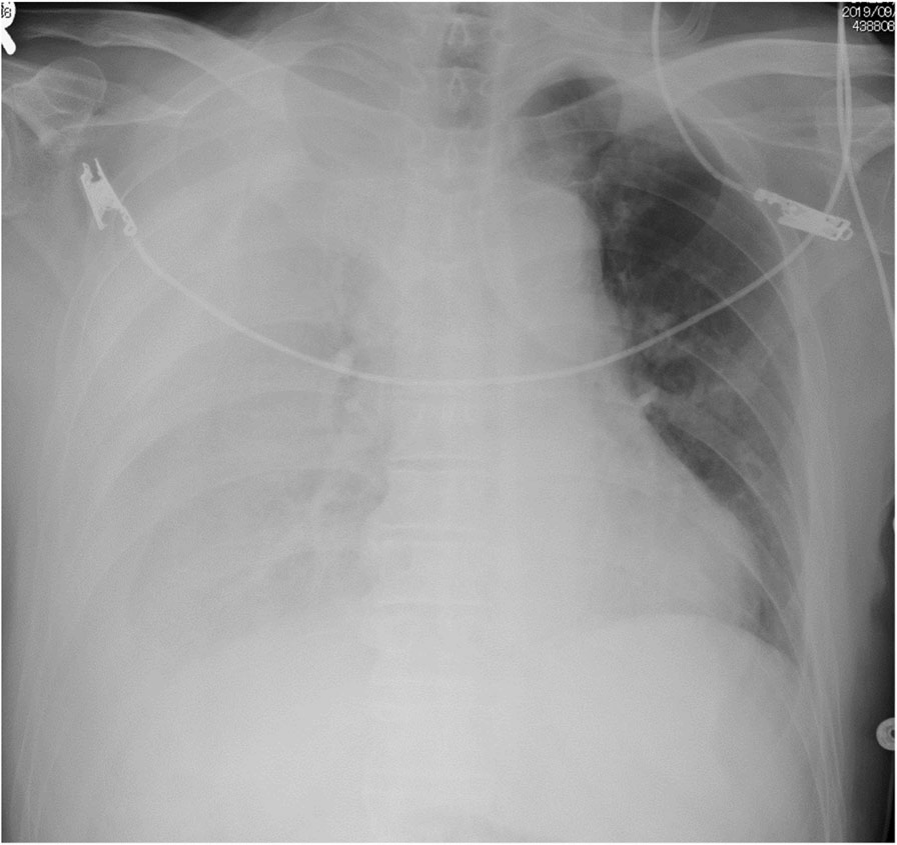
Chest X-ray showing decreased permeability of the right lung field

**Fig. 2:**
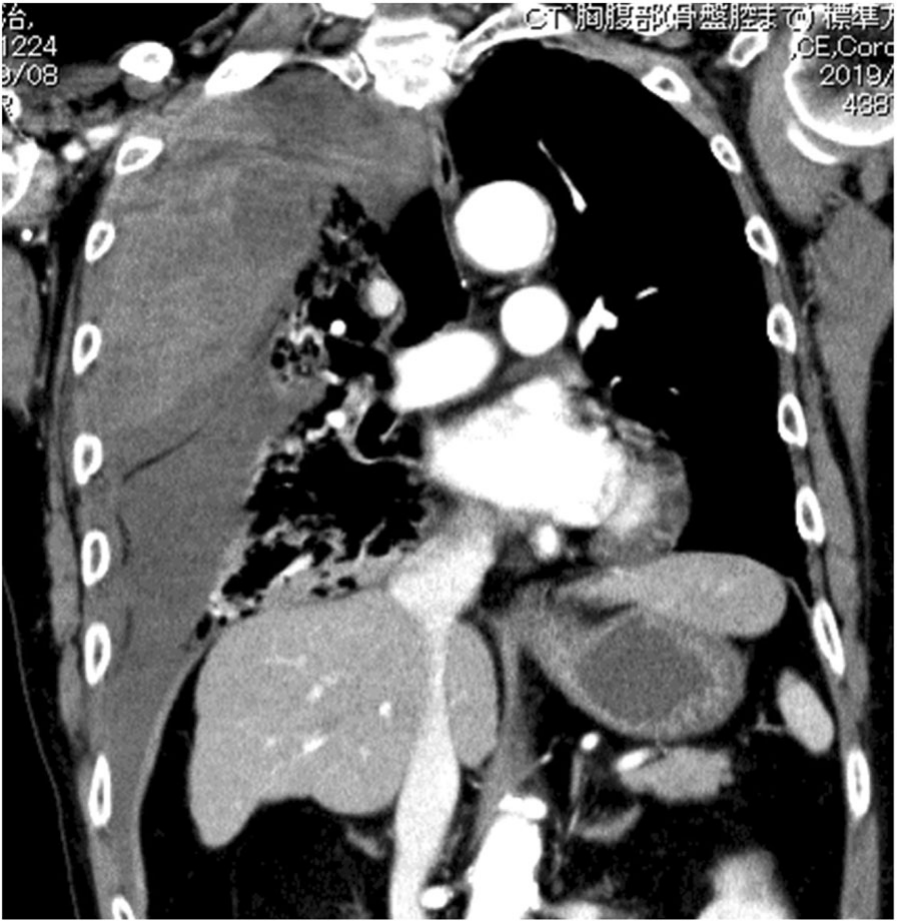
Chest computed tomography (CT) scan showing a hematoma in the extrapleural space

Chest radiography revealed massive right hemothorax (Fig. 1). Computed tomography (CT) of the chest revealed the presence of a huge lens-like encapsulated lesion measuring 220 × 90 mm in the right thoracic cavity (Fig. 2). Hemorrhagic effusion was obtained on thoracocentesis, and the lesion was suspected of being a hematoma. A coronary slice of reconstructed CT showed a well-defined, huge mass pressing against the normal lung tissue caudally. These findings all supported a diagnosis of extrapleural hematoma with hemothorax. A thoracic artery angiogram showed no apparent hemorrhaging at the aorta, right subclavian artery, or intercostal arterial branches.

**Fig. 3:**
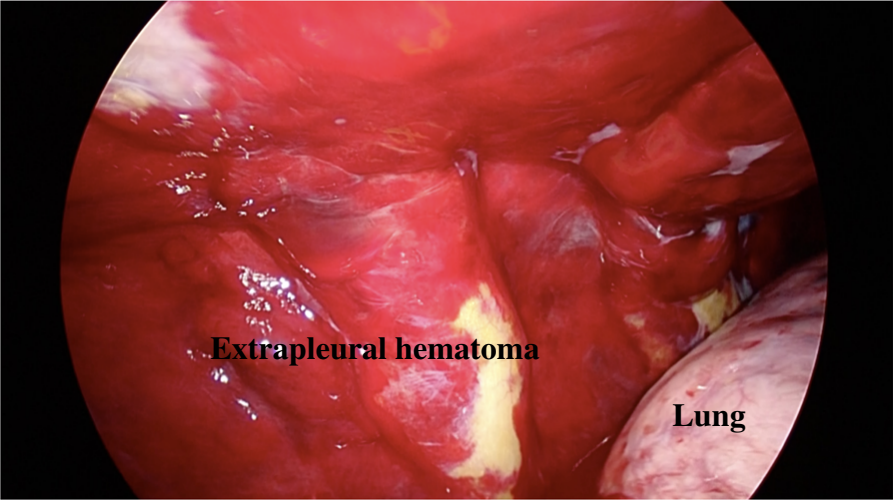
Operation findings showed an extrapleural hematoma

We performed video-assisted thoracic surgery (VATS). A huge hematoma was found in the extrapleural space (Fig. 3), and about half of it was unclotted. The hematoma was evacuated as extensively as possible, and the findings suggested that the hemorrhaging had been caused by intercostal artery injury. Therefore, we performed soft coagulation for the bleeding points and were eventually able to stop the bleeding. We then performed jet cleaning of the right thoracic cavity. The lung reexpanded almost fully, and his postoperative course was uneventful. We carefully search another bleeding point in thoracic cavity and confirm there was no bleeding using thoracoscopy before the chest was closed. The patient was discharged from our hospital on postoperative day 12.

### Case 2

A 73-year-old man was brought to our hospital by ambulance after bruising his back in his house. He complained of pain at his left back. His blood pressure was normal, oxygen saturation was 96%, and hemoglobin was 9.0 g/dl. His medical history included arteriosclerosis obliterans and bypass surgery, after which he had been taking an antiplatelet agent (aspirin).

**Fig. 4:**
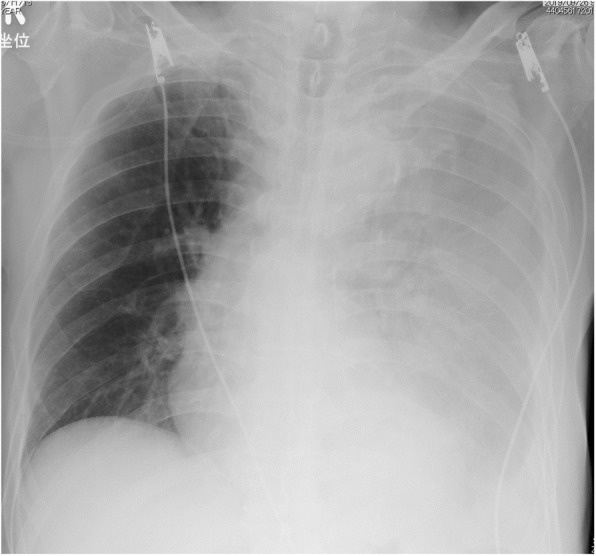
Chest X-ray showing decreased permeability of the left lung field

**Fig. 5:**
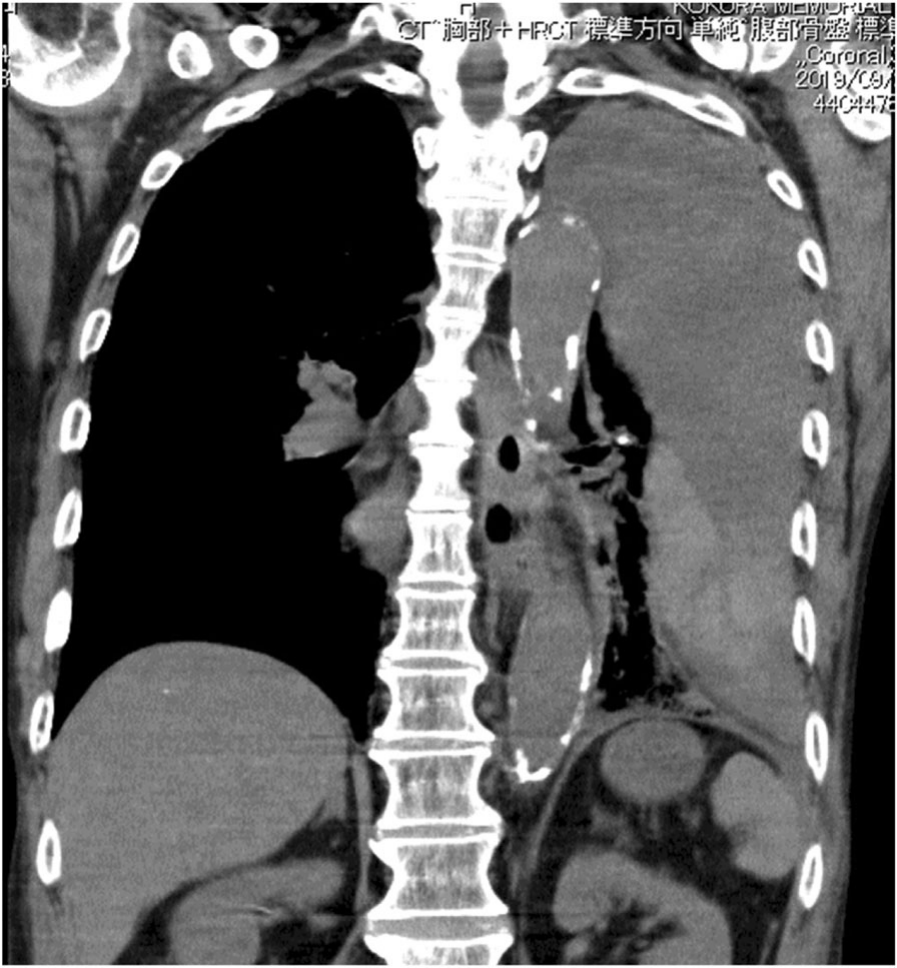
Chest computed tomography (CT) scan showing a hematoma in tt extrapleural space

Chest radiography revealed massive left hemothorax (Fig. 4). CT of the chest revealed the presence of a huge lens-like encapsulated lesion measuring 230 × 70 mm in the left thoracic cavity (Fig. 5). Hemorrhagic effusion was obtained by thoracocentesis, and the lesion was suspected of being a hematoma.

We performed VATS. The intrathoracic findings were similar to those of case 1. A huge hematoma was found in the extrapleural space, and about half of it was unclotted. We located a bleeding intercostal vessel near the broken 10th rib. Therefore, we performed soft coagulation for this bleeding point and were able to stop the bleeding. We carefully search another bleeding point in thoracic cavity and confirm there was no bleeding using thoracoscopy before the chest was closed. His postoperative course was uneventful. The patient was discharged from our hospital on postoperative day 7.

## Comment

This report has three important implications: First, extrapleural hematoma is empirically rare. It is defined as the presence of blood in the extrapleural space between the parietal pleura and the endothoracic fascia and is created when the blood cannot escape into the pleural cavity because the parietal pleura is still intact [3]. It has been reported that extrapleural hematoma can be caused by chest trauma or iatrogenic complications during central venous catheterization, rupture of an aneurysm of the thoracic aorta, a pleural biopsy, or even spontaneous rib fracture [2]. The diagnosis of extrapleural hematoma can be confirmed by chest radiography. Its typical finding is D-shaped opacity with the base located against the corresponding part of the chest wall [4]. The management of extrapleural hematoma is usually conservative. Poyraz et al. [2] recommended medical therapy or simple observation when the vital signs of an afflicted patient were stable and the hematoma was small; alternatively, surgical treatment was considered to be required if the hematoma was huge, causing circulatory and respiratory disturbances, or if the condition of the patient was unstable because of active bleeding.

Second, VATS was minimally invasive and effective in both of our cases. Sumida et al. reported a case of a huge extrapleural hematoma in an anticoagulated patient without trauma [5] and suggested that VATS might be effective as the first choice for managing extrapleural hematoma. Poyraz et al. [2], by contrast, recommended thoracotomy, and Rashid [6] suggested that an extrapleural hematoma is a relative major contraindication to VATS because of the poor visualization, as the extrapleural space is not a cavity anatomically. The best approach for managing an extrapleural hematoma is therefore controversial. VATS was indeed effective in both of our cases, but the position of the first port is important. The first port should be in the thoracic cavity. A good field of view cannot be achieved if the first port is placed in the hematoma itself. We performed three ports VATS. We checked for extrapleural hematoma from the thoracic cavity and then made an incision into the parietal pleura. We then removed the extrapleural hematoma. After that, we could find bleeding point and stop the bleeding using a soft coagulation system.

On that note, third, a soft coagulation system was useful for stopping bleeding. Sato reported that a soft coagulation system is a novel electrosurgical device that automatically maintains an output voltage below 190 V, resulting in pure coagulation without carbonization. Soft coagulation can be achieved with bipolar and monopolar devices in thoracic surgery. Bipolar scissors can be used to dissect pulmonary vessels safely and efficiently without damaging the vessel wall. Monopolar soft coagulation can be used to reduce bullous changes in the lung and stop air leakage from the lung parenchyma or bleeding from pulmonary vessels [7]. The bleeding point in both of the present cases were intercostal arteriovenous vessels, so we were able to stop the bleeding easily using soft coagulation.

## Conclusions

We reported two rare cases of extrapleural hematoma. This disease requires close attention when it manifests in patients undergoing anticoagulation therapy. In case 1, this patient had no apparent history of trauma. However, he had been taking antiplatelet agents. Therefore, we thought that the intercostal artery may have failed even with a slight impact. Regarding treatment, VATS was minimally invasive and particularly effective in these cases.

## Data Availability

Not applicable

## Abbreviations

CT: Computed tomography
VATS: Video-assisted thoracic surgery
